# The efficacy and safety of *Laminaria japonica* for metabolic syndrome: A protocol for systematic review

**DOI:** 10.1097/MD.0000000000028892

**Published:** 2022-02-18

**Authors:** Bonglee Kim, In-Seon Lee, Seok-Jae Ko

**Affiliations:** a Department of Pathology, College of Korean Medicine, Kyung Hee University, Seoul, Republic of Korea; b Department of Meridians and Acupoints, College of Korean Medicine, Kyung Hee University, Seoul, Republic of Korea; c Acupuncture & Meridian Science Research Center, Kyung Hee University, Seoul, Republic of Korea; d Department of Gastroenterology, College of Korean Medicine, Kyung Hee University, Seoul, Republic of Korea.

**Keywords:** Laminaria japonica, metabolic syndrome, randomized controlled trial, systematic review

## Abstract

**Background:**

Metabolic syndrome is a pathological condition characterized by abdominal obesity, insulin resistance, hypertension, and hyperlipidemia. Conventional treatments for metabolic syndrome have limitations due to their nonselectivity, drug resistance, and low bioavailability. The brown seaweed *Laminaria japonica* (LJP) is a traditional medicine and food in Asia that has shown pharmacological and biochemical properties favorable to the prevention and treatment of lifestyle-related diseases. We will systematically review randomized controlled trials and in vivo preclinical studies evaluating the efficacy and safety of LJP as a useful treatment for metabolic syndrome.

**Methods:**

The following databases will be searched from inception to September 2021: MEDLINE (via PubMed), EMBASE, Cochrane Central Register of Controlled Trials, and Web of Science. Randomized controlled trials and in vivo preclinical studies that analyzed LJP for the prevention and treatment of metabolic syndromes will be included. The outcome measures will include body composition, dietary habit scores, serum lipid profiles, daily nutrient intake, quality of life, number of microbiomes, gastrointestinal symptoms, and bowel function. Studies comparing LJP with any type of control intervention will be included. Data extraction using Review Manager version 5.3 and risk of bias assessment using the Cochrane Collaboration's tool for assessing risk of bias will be performed by 2 independent assessors.

**Results and Conclusion:**

This systematic review will provide evidence confirming the efficacy and safety of LJP in the treatment of metabolic syndrome.

**Ethics and dissemination:**

Ethical approval was not required, as this study protocol does not include any personal information of the participants.

**Trial registration number:**

DOI 10.17605/OSF.IO/G2BQK (https://osf.io/g2bqk).

## 1 Introduction

Metabolic syndrome, also known as syndrome X, is a pathological condition characterized by abdominal obesity, insulin resistance, hypertension, and hyperlipidemia.^[1]^ It is caused by impaired metabolic homeostasis in the human body.^[2]^ This disease is becoming increasingly common; for example, nearly 2 billion adults and one-third of all children worldwide are obese.^[3]^ This modern disease and its associated disorders have become a major global health problem.^[1]^ The causes of this growing metabolic syndrome patient population are associated with the increase in high-calorie, low-fiber food consumption and decrease in physical exercise.^[4]^

Conventional treatments for metabolic syndrome have limitations in terms of nonselectivity, drug resistance, and bioavailability.^[^5^,^^6^^]^ Novel candidates for use in treatment of metabolic syndrome are needed for drug development.

Recently, marine bioactive substances have attracted extensive attention and have been actively studied for potential use in the development of drugs and functional foods.^[7]^ The brown seaweed *Laminaria japonica* (LJP), also known as kelp, sea tangle, *kombu* or *kunbu* in Japanese and *Dashima* in Korea, is a traditional medicine and food in Asia because of its pharmacological and biochemical properties.^[^8^,^^9^^]^ LJP containing various bioactive compounds, such as proteins, iodine, vitamins, and polysaccharides, has shown efficacy in the prevention and treatment of lifestyle-related diseases, including hyperlipidemia,^[10]^ obesity,^[11]^ cancer,^[12]^ and others.^[13]^

In the present study protocol, we will systematically review randomized controlled clinical and experimental studies to summarize the current evidence on the efficacy and safety of LJP for the treatment of metabolic syndrome.

## 2 Methods and analysis

This systematic review protocol has been registered in the Open Science Framework registry (https://osf.io/g2bqk). This protocol will be performed in accordance with the preferred reporting items for systematic reviews and meta-analyses. Ethical approval is unnecessary because this is a literature-based study.

### 2.1 Inclusion and exclusion criteria for study selection

#### 2.1.1 Types of studies

The protocol for this systematic review will include randomized controlled trials, quasi-randomized controlled trials, and in vivo animal studies. Non-English studies, original studies (eg, case reports, reviews, commentaries), studies that are not related to LJP or metabolic syndrome, and in vitro studies will all be excluded. There is no restriction on the publication date.

#### 2.1.2 Characteristics of subjects

Patients with metabolic syndrome, also known as Reaven syndrome,^[14]^ syndrome X,^[15]^ and insulin resistance syndrome,^[16]^ will be included regardless of age, sex, or race. Patients will be included based on the 5 criteria proposed by the National Cholesterol Education Program-Adult Treatment Panel III (2001).^[17]^ The ATP III definition for metabolic syndrome indicates that patients should have 3 or more of the following conditions: central obesity: waist circumference >102 cm (male), >88 cm (female); hypertriglyceridemia: triglycerides ≥1.7 mmol/L; low high-density lipoprotein (HDL) cholesterol: <1.0 mmol/L (male), <1.3 mmol/L (female); hypertension: blood pressure ≥135/85 mm Hg or medication; and fasting plasma glucose ≥6.1 mmol/L. Subjects satisfying more than 1 of the 5 conditions for metabolic syndrome and healthy subjects included in investigations analyzing the effect of LJP on waist circumference, triglycerides, HDL cholesterol, blood pressure, or glucose will be included as supplementary data. Secondary pathologies originating from metabolic syndromes, such as chronic renal failure will be excluded. In in vivo animal studies focusing on diseases constituting metabolic syndromes, such as atherosclerosis, hyperlipidemia, obesity, and diabetes, will be included.

#### 2.1.3 Types of interventions

Randomized studies on LJP, kelp, sea tangle, *kombu*, and *kunbu* will be included. Modified LJP, such as water-extracted, roasted, or fermented LJP and LJP harvested at a given season (eg, *Harudori*-kombu which are young *kombu* harvested in the spring) or at a specific age will also be included. Seaweed or brown algae other than LJP, such as *Ascophyllum nodosum* and *Sargassum hemiphyllum*, will be excluded. Studies comparing LJP with any type of control intervention will be included. Animal studies using LJP or active compounds of LJP, such as fucoidan, polysaccharides, or polyphenols, will be included.

#### 2.1.4 Types of outcome measures

The outcome measures will include body composition assessments, dietary habit scores, serum lipid profiles (eg, triglycerides, HDL cholesterol, and low-density lipoprotein cholesterol), daily nutrient intake, quality of life assessment, number of microbiomes, gastrointestinal symptoms, and bowel function. Laboratory tests for malondialdehyde, catalase, superoxide dismutase, and glutathione peroxidase activities will be included. Animal studies using other serum tests (eg, insulin, amylin, or α-glucosidase) and assessments of cytokines (eg, interleukin-6, interleukin-10, or tumor necrosis factor-α) will be included.

### 2.2 Data sources

The following databases will be searched from inception to September 2021: MEDLINE (via PubMed), EMBASE, Cochrane Central Register of Controlled Trials, and Web of Science. The search terms will comprise parts of the disease term (eg, metabolic, Reaven, and syndrome X) and the intervention term (eg, LJP, kelp, *kombu* and *kunbu*). The search strategies designed for MEDLINE (via PubMed) are presented in Table 1. Modified search strategies will be applied to the other databases. Only studies published in the English language will be included.

**Table 1:** Search terms for MEDLINE via PubMed.

#1. *Laminaria japonica* or kelp or *kombu* or *kunbu*
#2. Metabolic or Reaven or syndrome X
#3. Cardiovascular or hypertension or HTN or blood pressure
#4. Glucose or diabetes or hyperglycemia or insulin resistance
#5. Triglyceride or HDL or cholesterol or dyslipidemia
#6. Waist circumference or fat or overweight or body weight or obes^∗^
#7. #2 or #3 or #4 or #5 or #6
#8. #1 and #7

### 2.3 Data collection and analysis

#### 2.3.1 Selection of studies

**Figure 1:**
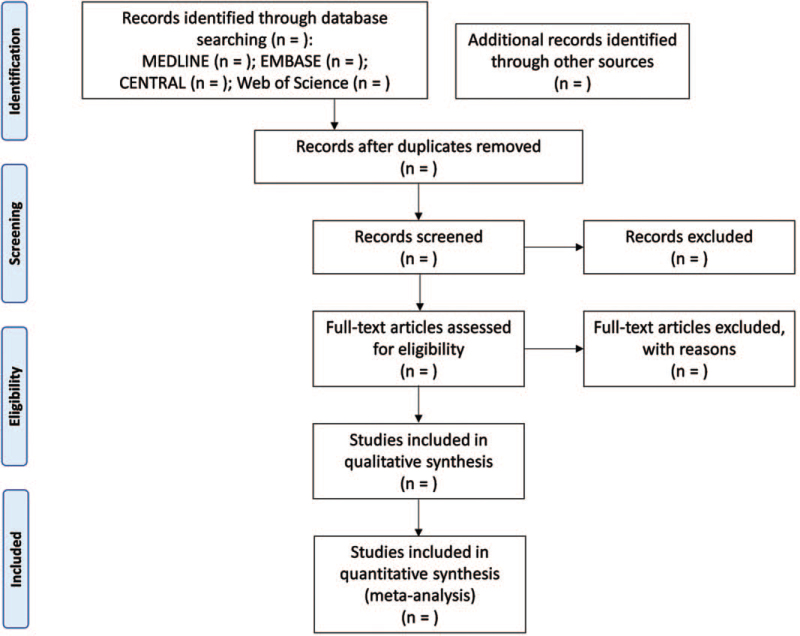
Flowchart for the process used in the database search.

Two reviewers (BK and IL) will independently review the titles, abstracts, and full text of the studies for eligibility for inclusion in this systematic review. All studies searched by electronic databases and identified by hand will be organized in Endnote X7 (Thomson Reuters, New York, NY). The reasons for excluding studies will be recorded and shown in the preferred reporting items for systematic reviews and meta-analyses flowchart (Fig. 1). Any disagreement between the 2 reviewers (BK and IL) will be resolved by discussion and consensus. If necessary, the arbiter (SK) will intervene and resolve any disagreement.

#### 2.3.2 Data extraction and management

Two review authors (BK and IL) will independently extract the data and fill out the standard data extraction form, which includes study information such as the first author, publication year, title, journal name, research design, number of patients, inclusion criteria, interventions, control, treatment period, outcome measures, main results, and adverse events. The following information and data will be extracted from animal studies: animal models, disease-inducers, positive controls, active compounds, administration route, dosage, treatment duration, mechanism, laboratory test results, efficacy of LJP, and reference. Any discrepancies will be resolved by discussion between the 2 reviewers (BK and IL), and if necessary, an arbiter (SK) will intervene to resolve the issue.

#### 2.3.3 Assessment of risk of bias in the included studies

Two reviewers (BK and IL) will evaluate the risk of bias based on the Cochrane Collaboration Tool,^[18]^ and the following design-based aspects of the studies will be included in the evaluation: random sequence generation (selection bias), allocation concealment (selection bias), blinding of participants and personnel (performance bias), blinding of outcome assessment (detection bias), incomplete outcome data (attrition bias), selective reporting (reporting bias), and other potential sources of bias. The results of the assessment will be presented in 1 of 3 categories: low, unclear, and high. Any disagreement between the 2 reviewers will be resolved through discussion, and if necessary, an arbiter (SK) will intervene.

#### 2.3.4 Measurement of treatment effects

We will use the relative risk with a 95% confidence interval to assess dichotomous data and the mean difference with a 95% confidence interval to evaluate continuous data.

#### 2.3.5 Unit of analysis

When crossover trials are reviewed, we will use only the first-phase data to avoid carry-over effects. When the trials have multiple intervention groups, a pairwise comparison will be performed.

#### 2.3.6 Dealing with missing data

We will attempt to contact the original author by email if there are any missing data. Statistical analysis will be performed using the intent-to-treat principle. If missing data cannot be obtained, the last observation carried forward method will be applied.

#### 2.3.7 Assessment of heterogeneity and publication bias

A random effects model will be used for the meta-analysis. We will assess heterogeneity using the I^2^ statistic, with I^2^ ≥ 50% indicating substantial heterogeneity. We will also evaluate heterogeneity using the χ^2^ test, with *P* < .1 indicating heterogeneity. In cases of heterogeneity, we will perform subgroup or sensitivity analyses to compensate for the heterogeneity. If there are more than 10 studies in the meta-analysis, we will investigate publication bias using a funnel plot.

#### 2.3.8 Data synthesis

Statistical analyses will be performed using the Review Manager program (v5.3, Nordic Cochrane Center, Copenhagen, Denmark; Cochrane Collaboration, 2014). Studies will be synthesized based on patient and outcome variables. If there are an insufficient number of studies to synthesize the data, we will review these studies. In the case of animal studies, we will describe the results in parallel and synthesize their mechanisms.

#### 2.3.9 Subgroup and sensitivity analyses

Subgroup analysis will be performed if there are sufficient subgroup studies to evaluate the cause of heterogeneity. The subgroup analysis criteria will include compounds of LJP, extraction methods, forms of LJP, control type, and duration of treatment. Low quality of studies can be excluded to test the robustness of the results. When there are sufficient studies, we will conduct a sensitivity analysis to assess the robustness of studies based on the sample size, quality of the method, and missing data.

#### 2.3.10 Grading the quality of evidence

We will use the grading of recommendations assessment, development, and evaluation approach to investigate the quality of evidence. Two researchers (BK and IL) will assess the level of quality of evidence as 1 of 4 rankings (high, moderate, low, or very low) according to downgrading factors, such as the risk of bias, inconsistency, indirectness, imprecision of the results, and publication bias, and upgrading factors, such as a large effect and dose-dependent response.^[19]^

## 3 Discussion

Metabolic syndrome is a cluster of metabolic disorders, such as hyperglycemia, insulin resistance, dyslipidemia, and obesity. It increases the risk of atherosclerotic cardiovascular disease and type 2 diabetes mellitus, leading to impairment of the quality of life of patients and increased mortality.^[20]^ The global epidemic proportion of metabolic syndrome is estimated to be 12% to 25%,^[1]^ and cardiovascular disease-related mortality is estimated to reach 22.2 million in 2030.^[21]^ The conventional treatment for metabolic syndrome and its comorbidities has not been satisfactory because of its low efficacy, chronic administration, and associated side effects.^[21]^ To prevent lifestyle-related diseases, researchers have paid attention to some of so-called “functional foods”, such as brown seaweed. LJP, a representative brown seaweed, is rich in healthy ingredients used for food preparation, including polysaccharides, fibers, and secondary metabolites (eg, polyphenols and fucoidans).^[^22^–^24^]^ LJP also has been demonstrated to exert multiple beneficial effects, including anticancer, antiviral, antioxidant, immunomodulatory, and anti-inflammatory effects.^[8]^ In this review, we provide an updated systematic review based on the most recent evidence from in vivo preclinical and clinical studies analyzing the potential of LJP to be used in the prevention and treatment of metabolic syndrome and its related comorbidities.

## Author contributions

Approved the final manuscript: All authors.

**Conceptualization:** Bonglee Kim, In-Seon Lee, Seok-Jae Ko.

**Data curation:** Bonglee Kim, In-Seon Lee.

**Funding acquisition:** Bonglee Kim.

**Investigation:** In-Seon Lee.

**Methodology:** Bonglee Kim, In-Seon Lee, Seok-Jae Ko.

**Supervision:** Seok-Jae Ko.

**Writing – original draft:** Bonglee Kim, Seok-Jae Ko.

**Writing – review & editing:** Seok-Jae Ko.
